# How to Give Medicine to Children

**Published:** 1889-04-06

**Authors:** C. R. Illingworth


### How to Give Medicine to Children.

By C. R. Illingworth, M.D.

"Noses have they, but they smell not" (Psalm cxv., 6).

"All in whose nostrils was the breath of life, of all that was in the dry land, died" (Genesis vii., 22).

The foregoing texts of scripture plainly indicate that, from the earliest times, the chief functions discharged by the nose were known to be those of smelling and breathing. It was only as the science of medicine advanced, and the compounding of nauseous drugs became better known, that a new use was found for this pronounced feature in the physiognomy of the younger members of the *genus homo*, more especially when vigorously refusing to imbibe a sufficient quantity of what was considered by the faithful disciples of Æsculapius to be essentially necessary for their health and life. This new use was that of a mouth-opener. The "breath of life" is of no use hovering round the exterior of the body, and as soon as the natural channel for it, through the nostrils, is blocked by firm and steady pressure with the finger and thumb, the mouth, it is averred, opens, more or less widely, for the life-giving breath. The champions of this ancient doctrine seize this opportunity for dashing in the nauseous or objectionable dose, the nostrils being firmly held until either the act of deglutition is in some way performed, or the whole is spat out. It is obvious, therefore, that the only service rendered by the closing of the nostrils is the opening of the mouth. But this is not always effected, for some children, promptly grasping the situation, will persistently breathe through the clenched teeth.

Now, it requires no argument to prove the unpleasant and even painful effect of swallowing by this process. Anyone by direct experiment will find that it gives rise to a feeling of tension in the ears, the result of the forcible driving of the current of air along the Eustachian tubes.

A study of the nervous mechanism of the processes of respiration and deglutition, however, should serve to point out the harm resulting from the violent obstruction offered to either. Both of these processes are carried on by muscles which are supplied by branches of the same nerve—the pneumo-gastric—and even in adults it is possible to have the two processes so confounded in the mind as to lead to a hysterical affection in the shape of a want of co-ordination of the movements of the pharynx and larynx, the patient breathing when swallowing should occur or is intended, and *vice versâ*.

But a study of the mechanism of deglutition itself, further, and in a much more pronounced degree, indicates the injurious nature of this practice. It is a physiological law that for voluntary muscular actions there must be fixed points. For instance, when the forearm is to be bent upon the upper arm at the elbow, the shoulder must be fixed for the origins of the biceps to take firm hold before the contraction of that muscle, acting upon its insertion into the radius, can be of any service in effecting the movement. In the same way, the muscles concerned in deglutition required fixed points before they can efficiently contract upon the morsel to be swallowed. Above and behind, the superior pharyngeal constrictor muscle has the base of the skull as a *punctum fixum*; but it is to the anterior portion that attention should most particularly be directed, because it is here that emphatic evidence is furnished of the absolute necessity for observing the natural process of deglutition before devising and recommending artificial substitutes for that important function, either in whole or in part. It is found, then, that the superior constrictor of the pharynx has attachments to the tongue, to the jaw, to the lower third of the internal pterygoid plate, and to the pterygo-maxillary ligament, which separates it from the buccinator (or cheek) muscle (these attachments can readily be seen on reference to any anatomical plates). Now note the mechanism of natural deglutition. The lips and jaws are firmly closed, and the tongue thrust against the hard palate, before the morsel or liquid to be swallowed is forced back into the embrace of the pharyngeal muscles. This closing of the jaws, lips, and cheeks for the fixation of the anterior attachments of the superior constrictors can be dispensed with, provided the tongue is fixed against the palate, but without a fixed point, provided either by that organ, by the lips and cheeks, or by the jaws, deglutition is impossible.

Now, picture a child of tender years and nervous temperament it may be, with the nose tightly nipped, preventing natural respiration, its mouth full of medicine, and the natural fixation of the anterior attachments of the pharyngeal muscles prevented by a spoon, and the danger of confusion in the child's mind between the processes of respiration and deglutition will be at once apparent. The result very frequently is either the suction of the medicine into the larynx by a violent inspiratory effort, or the forward forcing of it by an expiratory effort into the nose and Eustachian tubes. If the spoon be withdrawn, the child is just as likely to spit out the dose as to swallow it, in order to breathe again. Thus the only point gained by nipping the nose is the opening of the mouth, and if the practice stopped at that there would not be the same objection to its adoption.

But this is not the case. Moreover, there are better means, which at the same time subserve deglutition. A very young child has not learnt the use of the tongue, lips, and cheeks for the refusal of medicine, and it is not, as a rule, with such that trouble arises in its administration. Older children reject medicine either with the lips, the tongue, or the pharynx. By firmly pressing forward the cheeks, the child opens its mouth to cry and the lips are prevented approximating, whilst at the same time the pterygo-maxillary ligaments are fixed for the action of the superior constrictors. In very young children the medicine may then be poured gently into the mouth, and it will be easily swallowed. In older ones, when the tongue is used for the rejection of the dose, that organ can be pressed down by the back of the spoon, which then forms a fixed point for it instead of the arch of the palate. The third mode of rejection of the dose—that by gurgling with expiratory effort—may be prevented by the further introduction of the spoon and firm pressure on the back of the tongue.

The method of fixing the cheeks with the thumb and finger is thus advised, firstly, and chiefly, because it is a means of securing the first essential in deglutition; and secondly, because it leaves the natural respiratory channel unaffected, and thus prevents that terror arising from the confusion in the child's mind between the processes of respiration and deglutition, so commonly induced by nipping the nose.

